# A Critical Review of Dental Implant Materials with an Emphasis on Titanium *versus* Zirconia

**DOI:** 10.3390/ma8030932

**Published:** 2015-03-05

**Authors:** Reham B. Osman, Michael V. Swain

**Affiliations:** 1 Department of Oral Implantology and Prosthetic Dentistry, Academic Centrum of Dentistry Amsterdam (ACTA), 1081 LA Amsterdam, The Netherlands; 2 Department of Removable Prosthodontics, Cairo University, Giza 11553, Egypt; 3 Biomaterials Laboratory, Sydney Dental Hospital, the University of Sydney, Surry Hills, NSW 2010, Australia; E-Mail: michael.swain@sydney.edu.au

**Keywords:** zirconia, titanium, dental implants, oral implants, implant materials

## Abstract

The goal of the current publication is to provide a comprehensive literature review on the topic of dental implant materials. The following paper focuses on conventional titanium implants and more recently introduced and increasingly popular zirconia implants. Major subtopics include the material science and the clinical considerations involving both implant materials and the influence of their physical properties on the treatment outcome. Titanium remains the gold standard for the fabrication of oral implants, even though sensitivity does occur, though its clinical relevance is not yet clear. Zirconia implants may prove to be promising in the future; however, further *in vitro* and well-designed *in vivo* clinical studies are needed before such a recommendation can be made. Special considerations and technical experience are needed when dealing with zirconia implants to minimize the incidence of mechanical failure.

## 1. Introduction

Since their introduction by Brånemark in the 1960s, oral implants have become a reliable treatment option for the replacement of missing teeth [1]. The physical and chemical properties of implant materials are well-reported and documented factors that influence the clinical outcome and the prognosis of implant therapy. These properties include the microstructure of the implant, its surface composition and characteristics, as well as design factors [2]. An ideal implant material should be biocompatible, with adequate toughness, strength, corrosion, wear and fracture resistance [2,3]. The design principles of the implant should be compatible with the physical properties of the material. Materials used for the fabrication of dental implants can be categorized according to their chemical composition or the biological responses they elicit when implanted [4]. From a chemical point of view, dental implants may be made from metals, ceramics or polymers (Table 1).

**Table 1:** Materials used for the fabrication of endosseous dental implants.

Implant Material	Common Name or Abbreviation
I. Metals	
Titanium	CpTi
Titanium Alloys	Ti-6A1-4V extra low interstitial (ELI)
	Ti-6A1-4V
	Ti-6Al-7Nb
	Ti-5Al-2.5Fe
	Ti-15 Zr-4Nb-2Ta-0.2Pd
	Ti-29Nb-13Ta-4.6Zr
	Roxolid (83%–87%Ti-13%–17%Zr)
Stainless Steel	SS, 316 LSS
Cobalt Chromium Alloy	Vitallium, Co-Cr-Mo
Gold Alloys	Au Alloys
Tantalum	Ta
II. Ceramics	
Alumina	Al_2_O_3_, polycrystalline alumina or single-crystal sapphire
Hydroxyapatite	HA, Ca_10_(PO_4_)_10_, (OH)_2_
Beta-Tricalcium phosphate	β-TCP, Ca_3_(PO_4_)_2_
Carbon	C
	vitreous,
	low-temperature isotropic (LTI),
	ultra-low-temperature isotropic (ULTI)
Carbon-Silicon	C-Si
Bioglass	SiO_2_/CaO/Na_2_O/P_2_O_5_
Zirconia	ZrO_2_
Zirconia-toughened alumina	ZTA
III. Polymers	
Polymethylmethacrylate	PMMA
Polytetrafluoroethylene	PTFE
Polyethylene	PE
Polysulfone	PSF
Polyurethane	PU
Polyether ether ketone	PEEK

Adopted from: Williams, 1981 [5]; Lemons, 1990 [6]; Craig, 1993 [7]; Sagomonyants *et al.*, 2007 [8]; Berner *et al.*, 2009 [9].

The favourable long-term clinical survival rates reported for titanium and its biomedical alloys have made titanium the “gold standard” material for the fabrication of endosseous dental implants [10,11,12]. Occasionally, various metals and metal alloys involving gold, stainless steel and cobalt chromium have been used. However, adverse tissue reactions and a low success rate undermined their long-term clinical application and made these materials obsolete within the oral implant industry [4,13].

## 2. Titanium and Its Alloys

### 2.1. Physical and Mechanical Properties of Titanium and Its Alloys

According to the American Society for Testing and Materials (ASTM), there are six distinct types of titanium available as implant biomaterials. Amongst these six materials, there are four grades of commercially pure titanium (CpTi) and two titanium (Ti) alloys. The mechanical and physical properties of CpTi are different and are related chiefly to the oxygen residuals in the metal (Table 2). The two alloys are Ti-6Al-4V and Ti-6Al-4V-ELI (extra low interstitial alloys). The commercially pure titanium materials are called pure Grade I, Grade II, Grade III and Grade IV titanium. Commercially pure titanium is also referred to as unalloyed titanium and usually contains some trace elements of carbon, oxygen, nitrogen and iron. Theses trace elements markedly improve the mechanical properties of pure titanium [14] and are found in higher amounts from Grade I to Grade IV.

**Table 2:** Mechanical properties of commercially pure titanium and its alloys.

Material	Modulus (GPa)	Ultimate Tensile Strength (MPa)	Yield Strength (MPa)	Elongation (%)	Density (g/cc)	Type of Alloy
Cp Ti grade I	102	240	170	24	4.5	α
Cp Ti grade II	102	345	275	20	4.5	α
Cp Ti grade III	102	450	380	18	4.5	α
Cp Ti grade IV	104	550	483	15	4.5	α
Ti-6Al-4V- ELI	113	860	795	10	4.4	α + β
Ti-6Al-4V	113	930	860	10	4.4	α + β
Ti-6Al-7Nb	114	900–1050	880–950	8–15	4.4	α + β
Ti-5Al-2.5Fe	112	1020	895	15	4.4	α + β
Ti-15Zr-4Nb-2Ta-0.2Pd	94–99	715–919	693–806	18–28	4.4	α + β
Ti-29Nb-13Ta-4.6Zr	80	911	864	13.2	4.4	β

Adopted from: Lemons, 1990 [5]; Craig, 1993 [6]; Wataha, 1996 [4]; McCracken, 1999 [15].

Titanium alloys of interest to dentistry exist in three structural forms: alpha (α), beta (β) and alpha-beta. The alpha (α) alloys have a hexagonal closely packed (hcp) crystallographic structure, while the beta alloys (β) have a body-centred cubic (bcc) form. These different phases originate when pure titanium is mixed with elements, such as aluminium and vanadium, in certain concentrations and then cooled from the molten state. Aluminium is an alpha-phase stabilizer and increases the strength of the alloy, while it decreases its density. On the other hand, vanadium is a beta-phase stabilizer. Allotropic transformation of pure titanium (Ti) from the α to β phase occurs at 882 °C [15]. With the addition of aluminium or vanadium to titanium, the α-to-β transformation temperature changes over a range of temperatures. Depending on the composition and heat treatment, both the alpha and beta forms may coexist [3,14].

The alpha-beta combination alloy is the most commonly used for the fabrication of dental implants. This alloy consists of 6% aluminium and 4% vanadium (Ti-6Al-4V). Heat treatment of these alloys generating fine precipitation improves their strength, resulting in favourable mechanical and physical properties that make them excellent implant materials. They have a relatively low density, are strong and highly resistant to fatigue and corrosion. Although they are stiffer than bone, their modulus of elasticity is closer to bone than any other implant material, with the exception of pure titanium [12]. This lower modulus of elasticity is desirable, as it results in a more favourable stress distribution at the bone-implant interface [16].

Vanadium free α + β alloys, such as Ti-6Al-7Nb and Ti-5Al-2.5Fe, have been developed as implant materials because of toxicity concerns with vanadium. Furthermore, vanadium- and aluminium-free titanium alloys composed of non-toxic elements, like Nb, Ta, Zr and Pd, with a lower modulus of elasticity are under development. They are mainly β alloys that presumably, from a mechanical point of view, are more favourable compared to α + β alloys, because of their lower modulus of elasticity, which is closer to that of bone. The modulus of elasticity (E) of recently developed β-phase alloys is between 55 to 85 GPa, which is much lower than that of α + β alloys (113 GPa), yet still greater than that of cortical bone with a value ranging between 17 and 28 GPa and cancellous bone with E values between 0.5 and 3 GPa [17]. These alloys are also capable of attaining higher strength and toughness compared to α + β alloys [12].

Recently, a new alloy for manufacturing narrow diameter implants (Roxolid^®^, Straumann, Basel, Switzerland) has been introduced to dentistry [9,18]. The alloy is based on the binary formulation of 83%–87% titanium and 13%–17% zirconium [9,18]. It has been claimed that this alloy exhibits better mechanical characteristics compared to CpTi and Ti-6Al-4V with a tensile strength value of 953 MPa [18] and a fatigue strength value of 230 N, according to ISO 14801 internal tests (manufacturer’s information). The exact data on the elastic modulus of this material is still missing [19]. An *in vivo* biomechanical study in an animal model showed that the novel Ti-Zr alloy had significantly higher (*p* = 0.02) removal torque values (230.9 ± 22.4 Ncm) in comparison to Ti (204.7 ± 24 Ncm) [9].

### 2.2. Titanium Sensitivity Associated with Dental Implants

Recently, there have been some concerns that titanium might evoke an unwelcome host reaction; however, little evidence is available in the literature, and tentative judgments are only based on case studies [20] and isolated clinical reports [21]. A possible association between surface corrosion of titanium, on the one hand, and hypersensitivity reactions, on the other hand, has been discussed in the literature [22,23,24,25,26]. Following skin or mucosal contacts, metal ions will be released from implants, which then form complexes with native proteins and act as allergens, causing hypersensitivity reactions [27]. An increased concentration of titanium has been detected in peri-implant tissues, regional nodes and pulmonary tissues in animal models with failed implants [28]. Furthermore, a somewhat higher blood concentration of titanium has been described in patients with failed loose hip prostheses [29,30]. However, it is worth noting that the dental clinical relevance of these findings is not yet clear.

Allergy to titanium in the medical literature has been described in the form of urticaria, pruritus of the skin or mucosa, atopic dermatitis [31], impaired healing of fractures [32] and pain, necrosis and weakening of orthopaedic implants [33]. Non-specific immune suppression or overaggressive immune responses have also been reported as different forms of allergy to titanium, particularly with sensitive patients. However, whether the findings of these studies can be extrapolated to the oral cavity and dental implants is debatable [23]. One reason would be the smaller intraosseous contact surface of dental implants compared to orthopaedic implants, considering that bone has a very low reactivity potential [34]. On the other hand, the decreased permeability of mucosa compared to skin implies that the antigen concentration has to be 5–12-times greater to elicit the same response. Furthermore, the salivary glycoprotein layer formed on implant surfaces once implanted into the oral cavity may act as a protective barrier [35]. In the dental literature, titanium hypersensitivity has been described in the form of facial eczema, dermatitis, rashes, non-keratinized oedematous hyperplastic gingiva and rapid implant exfoliation, which could not be attributed to infection, impaired healing or overload [23,36,37,38,39]. A recent review suggested that the incidence of allergic reactions to titanium dental implants may be under-reported as a possible etiological factor in implant failure, due to lack of recognition and infrequent or ambiguous clinical presentations [25].

In summary, the clinical relevance of allergic reactions in patients with titanium dental implants remains debatable. The results of two recent reviews on the topic reported different conclusions [25,26]. In the first review, it was concluded that the significance of titanium as a cause of allergic reactions in patients with dental implants remains unproven [26]. On the other hand, the results of the other review indicated that titanium could induce hypersensitivity in susceptible patients and might play a critical role in causing implant failure [25].

### 2.3. Failure Mode of Titanium

**Figure 1:**
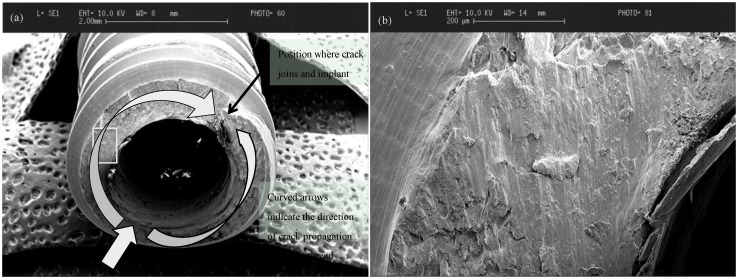
(**a**) Low magnification (×25) SEM image of fractured titanium implant. The crack in this instance was initiated on the lower left edge of the implant (lower straight arrow) and extended around the thread, finally breaking when the cracks overlapped on the upper right-hand side; (**b**) higher magnification (×500) view of the rectangular outlined area of the fractured surface in Figure 1a showing fatigue striations in a vertical pattern that mark the crack position as it progressed.

Titanium implant fracture is an uncommon occurrence with a reported incidence ranging from 0% to 6% [40,41,42,43,44]. Potential causes of implant fracture may be of three major categories: (i) implant design; (ii) manufacturing defects; or (iii) non-passive fit of the framework or physiological and biomechanical overload [45]. A retrospective study that analysed titanium implant fractures found the majority of fractures to be more common with 3.75-mm diameter implants made from commercially pure Grade I titanium, supporting partially edentulous restorations and proceeded with screw loosening [46]. Screw loosening may be caused by poor prosthodontic design, occlusal overload or parafunctional activities [47]. In the literature, the proposed mechanism of titanium implant failure is metal fatigue from high cyclic occlusal loading [48,49]. Accelerated peri-implant marginal bone loss is suggested to result in an increase in the bending moments and torque force on the implants. This, in turn, contributes to increased implant mobility and an eventual structural failure of the implant. Concurrently, the SEM analysis of fractured titanium implants reveals consistent uniformity of the microstructure with no indications that major inclusions or porosities are present and refutes the possibility of implant failure due to manufacturing errors [40,45]. All of the fractures were compatible with signs of fatigue failure that appeared to initiate from the stress concentration associated with the thread and typically initiated in the section of the implant that was not internally supported by the abutment screw; Figure 1a. The crack typically follows the thread, and when the crack has almost completely encircled the implant, a tearing-like fracture occurs, linking the cracks on adjacent threads. The complete reloading of the fracture surface of the implant under loading often plastically deforms the fracture surface, making classic fractographic surface analysis features difficult to discern. The image shown in Figure 1b in the outlined area of Figure 1a does show the presence of fatigue striations that are almost vertically aligned and constitute the progressive location of the crack front under cyclic loading as it progresses from left to right (Figure 1). However, till now, it has been unclear whether marginal bone loss is a cause or an effect of implant fracture or if they are both consequences of unfavourable loading [50]. From a chemical point of view, it has been suggested that titanium implants may adsorb hydrogen from the biological environment, consequently becoming more brittle and prone to fracture [51]. Furthermore, Green *et al.* suggested that galvanic corrosion between non-precious metal alloy restorations supported on titanium implants might initiate a cytotoxic reaction, as well as potentially assist with fatigue crack initiation. Such a reaction results in accelerated peri-implant bone resorption and subsequent increase in bending forces on implants with the eventual failure of the involved implants [47]. However, there is little evidence to support the chemical theory as a major contributor to implant failure [50].

## 3. Ceramics

### 3.1. Ceramics as Dental Implant Coatings

Ceramics were first introduced to implant dentistry in the form of coatings onto metal-based endosseous implants to improve osseointegration. Over the last 15 years, various forms of ceramic coatings have been used on dental implants [52] (Table 3). This involved the utilization of both bioactive ceramics, such as calcium phosphates and bioglasses, and inert ceramics, including aluminium oxide and zirconium oxide. Coatings can be dense or porous, with a thickness ranging from 1 to 100 μm, depending on the coating method that is employed. Different methods to coat metal implants comprise plasma spraying, sputter-deposition, sol-gel coating, electrophoretic deposition or biomimetic precipitation [52]. Bioactive ceramics have been shown to release calcium phosphate ions around the implants, resulting in enhanced bone apposition compared with the more inert ceramic and metallic surfaces [52,53,54,55,56]. Amongst the most popular calcium phosphate coating materials are plasma-sprayed dense hydroxyapatite and fluorapatite. These coatings, depending on the deposition conditions, contain regions that are partially amorphous; at the same time, they also retain regions that are highly crystalline. This appears to be important for eliciting specific biological responses to these materials. Furthermore, denser coatings are characterized by higher strengths and lower solubility. Inert ceramic materials are rarely used as coatings, because they do not appear to be as osteoconductive as the more bioactive calcium phosphate materials. Despite a clinical success of 97.8% reported for hydroxyapatite (HA)-coated implants, concerns about degradation and debonding of these coatings have been raised [57,58,59].

**Table 3:** Ceramic materials available as dental implants and coatings.

Material	Chemical Composition
Hydroxylapatite (HA)	Ca_10_(PO_4_)_6_(OH)_2_
Tricalcium phosphate (TCP)	α, β,Ca_3_(PO_4_)_2_
Fluorapatite (FA)	Ca_10_(PO_4_)_6_F_2_
Tetracalcium phosphate	Ca_4_P_2_O_9_
Calcium pyrophosphate	Ca_4_P_2_O_7_
Brushite	CaHPO_4_, CaHPO_4_·2H_2_O
Bioglasses	SiO_2_-CaO-Na_2_O-P_2_0_5_-MgO, *etc.*
Aluminium oxide	Al_2_O_3_
Zirconium oxide	ZrO_2_

Adopted from Lacefield, 1998 [52].

### 3.2. Ceramics as Dental Implant Materials

With the development of biomaterials science and industrial technology, interest in ceramics for dental application has been renewed. Ceramics, particularly the yttrium-stabilized tetragonal polycrystalline zirconia (Y-TZP), exhibit improved mechanical properties that make them suitable substrates for the fabrication of dental implants [60].

#### 3.2.1. Mechanical Properties of Zirconia

Zirconia holds a unique place amongst oxide ceramics due to its excellent mechanical properties [61]. Yttria stabilised tetragonal zirconia polycrystalline (Y-TZP) materials exhibits superior corrosion and wear resistance, as well as a high flexural strength (800 to 1000 MPa) compared to other dental ceramics [62,63]. An *in vitro* study reported the fracture strength of one-piece unloaded zirconia implants to be 512.9 N *versus* 410.7 N after artificial loading [64]. The effect of cyclic loading and preparation on the fracture strength of one-piece zirconia implants was also investigated. Kohal *et al.* found a decrease in fracture strength resistance following the cyclic loading and implant preparations, though with the values yet within the acceptable clinical levels to withstand average occlusal forces [65]. On the other contrary, Silva *et al.* found no influence of crown preparation on the reliability of one-piece zirconia implants at loads under 600 N [66]. On analysing the mechanical properties and reliability of two-piece zirconia implants, Kohal *et al.* reported low fracture strength values for both loaded and unloaded implants (average: 280 N) and accordingly could not recommend this implant prototype for clinical use [67]. At ambient pressure, unalloyed zirconia can assume three crystallographic forms, depending on the temperature. At room temperature and upon heating up to 1170 °C, the structure is monoclinic. It assumes a tetragonal form between 1170 and 2370 °C and a cubic structure above 2370 °C and up to the melting point. Alloying pure zirconia with stabilizing oxides, such as CaO, MgO, Y_2_O_3_ or CeO_2_, allows the retention of the metastable tetragonal structure at room temperature. Dental procedures, such as grinding or sandblasting, can trigger a tetragonal to monoclinic transformation in the surface region [63]. This transformation is accompanied by a substantial increase in volume (~4.5%) that induces surface compressive stresses, thereby closing the crack tip and enhancing resistance to further propagation. This characteristic, known as transformation toughening, increases the fracture strength and fracture toughness of Y-TZP ceramics compared with other dental ceramics [68]. On the contrary, increased phase transformation toughening may alter the phase integrity of the material and increases the susceptibility of the material to low-temperature degradation [69].

#### 3.2.2. Low Temperature Degradation

Low-temperature degradation (LTD), also known as ageing, occurs by a slow surface transformation of the metastable tetragonal crystals to the stable monoclinic structure in the presence of water or water vapour. As previously mentioned, a certain degree of surface tetragonal-monoclinic transformation can actually improve the mechanical properties of Y-TZP. However, a narrow range exists between improvement and destruction of mechanical properties, as further ageing results in property deterioration [70]. Transformation starts first within isolated grains on the surface by a stress corrosion mechanism. The transformation of one grain leads to a volume increase, thereby stressing the neighbouring grains and generating microcracking, which enables further water penetration, crack propagation and phase destabilization [71]. Experimental observations have shown that the degradation proceeds most rapidly at temperatures between 200 and 300 °C and is time dependent.

The ageing process depends on several microstructure features, such as porosity, residual stresses, grain size and the stabilizer content of the processed material. A decrease in grain size and an increase in stabilizer content were found to retard the transformation process. The critical grain size reported in the literature ranges from 0.2 to 1 μm depending on the Y_2_O_3_ content. A grain size larger than 1 μm was reported to exhibit a large amount of tetragonal-monoclinic transformation, together with a remarkable decrease in strength, whereas grains of <0.4 μm showed no significant change of phase content or strength. At a grain size below 0.2 μm, no tetragonal-monoclinic transformation can occur, resulting in reduction of fracture toughness. Watanabe *et al.* estimated that the critical grain size for tetragonal phase retention depends on Y_2_O_3_ content and suggested that the critical grain size increases from 0.2 to 0.6 μm as the Y_2_O_3_ content increases from 2 to 5 mol% [72].

LTD results in an adverse cascade reaction involving Y-TZP grain pull out, roughening of the surface, increased wear and microcracking. When the microcracked and damaged zone reaches the critical size for slow crack growth to proceed, degradation in mechanical properties of the material will occur. It was claimed that the ageing process was limited and not critical in the *in vivo* situation until the year 2001, when roughly 400 orthopaedic femoral heads failed in a very short period. Upon investigation of the reasons for failures of these heads, Chevalier found it to be associated with an accelerated ageing related to the changes in the processing techniques used. The latter led to changes in the microstructure of the Y-TZP material [71]. Thus, the author emphasized the importance of accurate processing techniques in avoiding accelerated ageing as compared to normal ageing processes. In accordance, clinical retrieval studies appear to confirm the strong variability of zirconia heads with regard to LTD resistance, supporting the claim that the inaccuracy in the processing techniques was the major causative factor that affects LTD resistance. However, the need for more advanced studies on the correlation between microstructure and LTD resistance *in vivo* is still warranted. An *in vitro* simulation investigating the effect of ageing on zirconium oxide used for oral rehabilitation found that although ageing reduces the mechanical features of zirconia, the decrease occurs within clinically acceptable values [73]. In accordance, the findings of a recent *in vitro* study showed that dental procedures did not significantly change the bulk properties of zirconia (flexural strength and yttria composition), indicating a longer-term *in vivo* function without biomechanical fracture [74].

Attempts to minimize LTD of 3Y-TZP systems include the addition of small amounts of silica [75], the use of yttria-coated rather than co-precipitated powder, the reduction of the grain size [76], an increase of the stabilizer content or even the formation of composites with aluminium oxide (Al_2_O_3_) [77]. A composite material processed with 80% tetragonal zirconia polycrystals (ZrO_2_-TZP) and 20% alumina (Al_2_O_3_) is reported to have outstanding mechanical and tribological properties. The addition of alumina to zirconia clearly hinders ageing or at least drastically reduces its kinetics, as it changes the grain-boundary chemistry and limits the tetragonal grain growth during sintering which results in a more stable structure [77].

#### 3.2.3. Failure Mode of Zirconia

**Figure 2:**
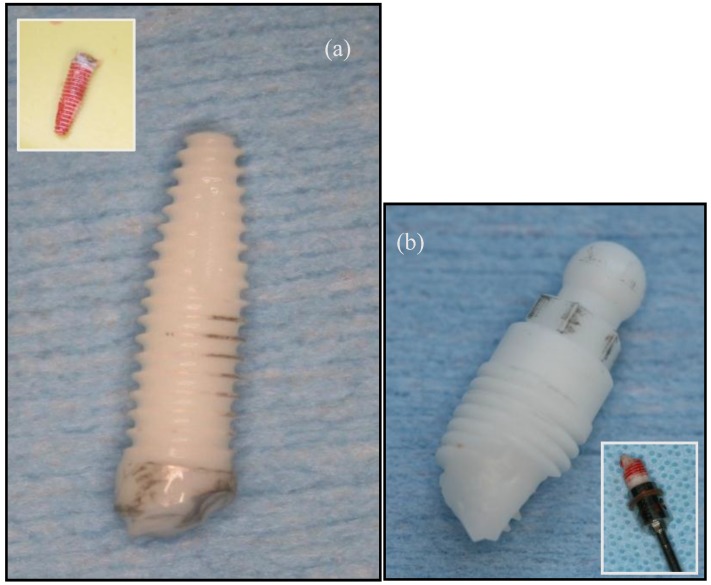

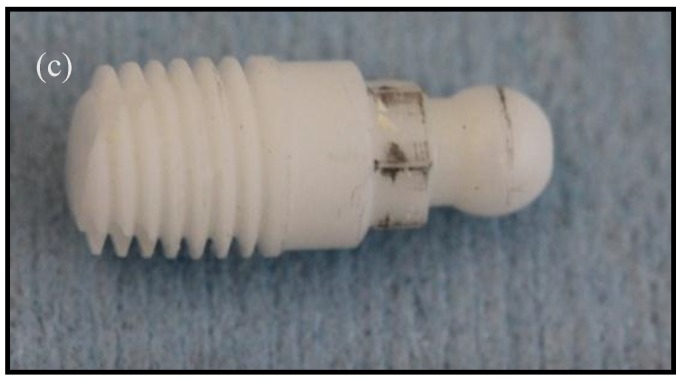
Clinical examples of fractured zirconia implants: (**a**,**b**) implants fractured during surgery; (**c**) implants fractured after prosthodontic loading.

An understanding of the biomechanical failure modes of zirconia implants is essential so that an optimal zirconia implant design can be developed [78]. Based on more fundamental studies in the biomaterials science field, the physical mechanism of ceramic implant failure can be either chemical and/or mechanical in nature [79]. Mechanical failure can occur either during the surgical placement of the implant [78] or subsequent functional loading [80] (Figure 2). 

**Figure 3:**
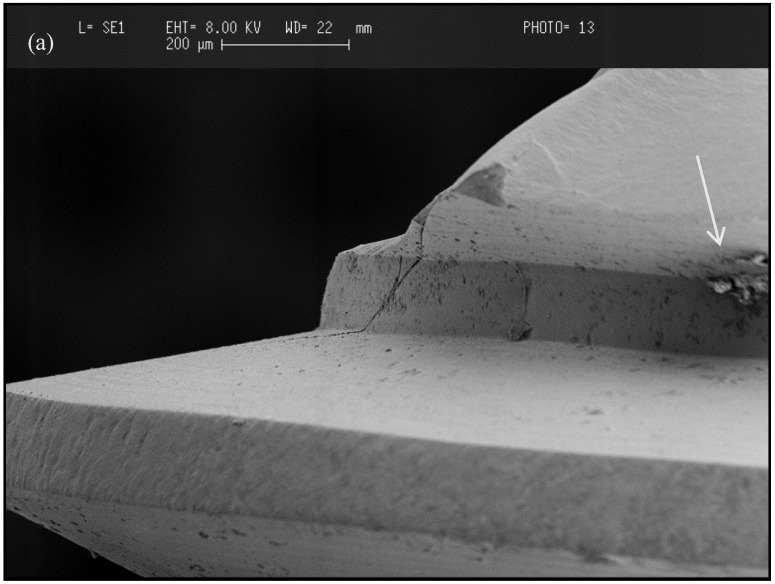

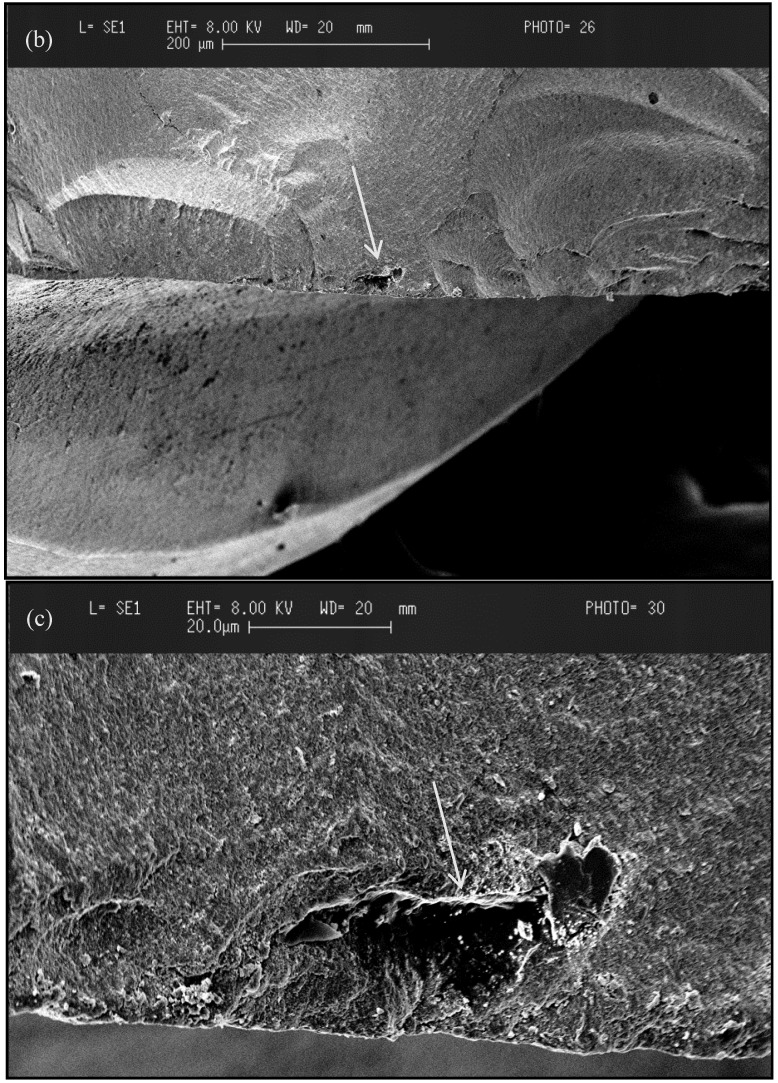
SEM images at different magnifications of various fractured zirconia implants showing porosities (white arrows) pointed out as the cause of implant fracture: (**a**) magnification factor: ×250; (**b**) magnification factor: ×500; (**c**) magnification factor: ×800.

Contrary to titanium implants, manufacturing imperfections or flaws created during ceramic implant fabrication and subsequent surface treatment may compromise their strength [78]. Material flaws usually assume the form of pores or microcracks of a submillimetre scale [79]. An example of a clinically fractured implant is shown in Figure 3, revealing a fracture originating from a pore entrapped within the implant body during the manufacturing process. Such cracks when combined with high bending moments or biomechanical overload can initiate crack propagation and result in early implant failure [80]. Bending load exerts a bending moment on the fixture cross-section at the crestal bone level. Peri-implant bone resorption increases the crown to implant ratio, resulting in an increase in bending moment induced forces and, combined with lateral occlusal loading, can result in premature implant failure [50].

**Figure 4:**
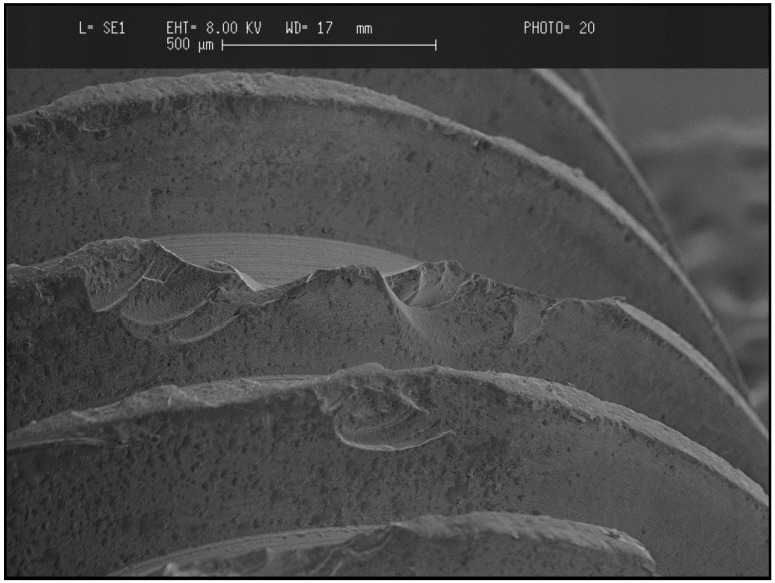
SEM image of failed retrieved zirconia implant showing the vulnerability of deep, thin, sharp thread edges easily fractured with a trephine bur.

Geometrical implant design can also be a contributing factor for ceramic implants failures. Considering the brittle nature of ceramics, all areas of excessive stress concentration should be avoided. This includes, but is not limited to, the configuration of the thread design. Sharp, deep and thin threads as well as sharp internal line angles represent areas of stress concentration that can enhance the likelihood of crack propagation and implant failure [78] (Figure 4). A reduced implant diameter of 3.25 mm, associated with a higher bending moment, has also been reported by Gahlert *et al.* to be a contributing factor for implant fracture during functional loading [80]. During surgical procedures, difficulties can be encountered when inserting the implants in dense hard-type bone. If hand torqueing is needed for final insertion of the implant and the applied forces are not purely rotational in nature, bending forces may be generated, resulting in implant failure [78]. LTD can account for the failure of zirconia implants through a process similar to “subcritical” or “slow-crack” growth (SCG), as described above [71,81]. However, whether the ageing of zirconia is of concern clinically still has to be shown. No clinical data on this topic can yet be found. It is in the authors’ opinion that the failure mechanism of zirconia implant occurs through an interaction of the above-described chemical and mechanical factors.

#### 3.2.4. Types of Zirconia Used in Dentistry

Despite the plethora of the zirconia-containing ceramic systems available on the market today, to date, only three have been used in dentistry. These are yttrium-stabilized tetragonal zirconia polycrystals (3Y-TZP), alumina-toughened zirconia (ATZ) and zirconia-toughened alumina (ZTA).

##### (i) Yttrium-Stabilized Tetragonal Zirconia Polycrystals (3Y-TZP)

The microstructure of 3Y-TZP ceramics for dental applications consists of up to 98% small equiaxed tetragonal grains (0.2–0.5 μm), sometimes combined with a small fraction of the cubic phase, and 3 mol% yttria (Y_2_O_3_) as a stabilizer. The mechanical properties of 3Y-TZP depend on the grain size, which is dictated by the sintering temperature [82]. Above a grain size of 1 μm, 3Y-TZP is less stable and more susceptible to spontaneous tetragonal to monoclinic transformation [83]. Below a grain size of 0.2 μm, transformation toughening is not possible, which results in reduced fracture toughness [84]. Higher sintering temperatures and longer sintering times lead to larger grain sizes.

Recently, 3Y-TZP has been used as a substrate for the manufacturing of single piece endosseous oral implants. Prosthetic restorations with 3Y-TZP are obtained by soft machining of pre-sintered blanks followed by sintering at high temperature or by hard machining of fully sintered blocks [85]. Soft machining of 3Y-TZP utilizes materials with final sintering temperatures varying between 1350 and 1550 °C, depending on the manufacturer [62,86]. This fairly wide range of sintering temperatures has an influence on the grain size and, later, the phase stability of 3Y-TZP for dental applications. The soft machining of zirconia blanks and subsequent high temperature sintering prevents the stress-induced transformation from tetragonal to monoclinic and leads to a final sintered surface virtually free of the monoclinic phase, unless grinding adjustments are needed or sandblasting is performed. On the other hand, hard machining of fully-sintered zirconia blocks has been shown to result in a significant amount of monoclinic phase with subsequent surface microcracking and higher susceptibility to low-temperature degradation [86]. Recently, introducing small amounts of alumina to 3Y-TZP has produced ceramic blocks known as TZP-A. The added traces of alumina improve the durability and stability under high temperatures and humid environments. However, this has been at the expense of the reduced translucency of ceramic blocks [86,87,88].

##### (ii) Glass-Infiltrated Zirconia-Toughened Alumina (ZTA)

Ceramics based on zirconia are combined with a matrix of alumina (Al_2_O_3_) to advantageously utilize the stress-induced transformation capability of zirconia and produce a structure known as ZTA (alumina reinforced with zirconia grains). The material is developed by adding 33 vol% of 12 mol% ceria-stabilized zirconia (12Ce-TZP) to In-Ceram alumina [87]. In-Ceram zirconia can be processed by either slip-casting or soft machining prior to glass infiltration [62]. The slip casting technique is associated with very limited amounts of shrinkage upon sintering. However, the considerable amount of residual porosity incorporated (8%–11%) during the procedure results in the lower mechanical properties of this material compared to 3Y-TZP dental ceramics [89,90,91]. On the other hand, the soft machining of the pre-sintered Y-TZP blocks results in higher mechanical properties, creating tougher prostheses, but with sintering contractions of around 25% [63].

##### (iii) Alumina Toughened Zirconia (ATZ)

ATZ is a composite ceramic material consisting of a mixture of 20 wt% alumina and 80 wt% zirconia containing 3 mol% yttria. The addition of alumina as low as 0.25 wt% to TZP ceramics significantly improves the resistance of the material to surface degradation (LTD). ATZ exhibits the highest bending strength known for ceramics, both at room temperature (1800–2400 MPa) and at elevated temperatures (>800 MPa at 1000 °C). Such high strength properties result in a high thermal shock resistance of ATZ ceramics as it prevents strength reduction on rapid cooling from elevated temperatures [92].

## 4. Osseointegration of Y-TZP *versus* Titanium Dental Implants

Animal studies demonstrated bone integration of threaded zirconia implants comparable to that of titanium after insertion in different animal models and sites and under different loading conditions. In those studies, the mean bone-implant contact was above 60%, indicating successful osseointegration. Akagawa *et al.* [93] were the first to evaluate loaded *versus* unloaded zirconia oral implants in beagle dogs. They found direct bone apposition to the implants in both groups with a bone-to-implant contact ratio of 81.9% and 69.8% for the unloaded and loaded groups, respectively. In a follow-up study, the same authors evaluated osseointegration of Y-TZP implants subjected to different loading modalities in monkeys [94]. No significant differences were detected in clinical parameters or osseointegration, nor were there any mechanical problems encountered between different loading groups. The different loading groups were a single implant, two connected implants and implant-tooth-supported restorations. However, in the previously mentioned studies, no titanium control group was included for comparison. In a split mouth design, Kohal *et al.* [95] compared osseointegration and peri-implant soft tissue dimensions between loaded titanium and zirconia implants in a primate model and found no statistical difference between the two materials. Several other animal investigations showed that zirconia implants undergo osseointegration similar to [96,97,98,99,100] or even better [101] than that of titanium implants.

A number of studies investigated the influence of surface microtopography on the osseointegration of zirconia implants. In a rabbit model, Sennerby *et al.* [102] and Rocchietta *et al.* [103] histologically and biomechanically analysed the bone tissue response to Y-TZP with different surface topographies and used oxidized titanium implants as controls. The removal torque values were significantly higher for surface-modified zirconia and titanium implants compared to machined-surface implants, with no significant difference regarding bone-to-implant contact between the two different materials. In accordance with previous results, Gahlert *et al.* [104] concluded that sandblast-roughened ZrO_2_ implants enhanced the bone stability and achieved a higher stability in the bone compared to machined-surface implants. Schliephake *et al.* [105] compared the peri-implant bone formation and mechanical stability of surface-modified zirconia implants with sandblasted and acid-etched titanium implants and found similar degrees of bone implant contact and bone volume density for all of the implants, despite the fact that the titanium surface was significantly rougher than the tested zirconia surfaces. However, titanium implants were found to have a higher removal torque resistance, probably due to the difference in the surface roughness.

A cell culture study by Bächle *et al.* [106] found that cell attachment and proliferation of osteoblast-like cells on Y-TZP disks of differently treated surfaces were comparable to those of a sandblasted/acid-etched titanium surface. In contrast, another study showed that modified zirconia surfaces mediate more pronounced adhesion, proliferation and differentiation of osteoblasts compared with titanium [107]. Another study investigated the osteoblast cell adhesion on laser-modified Y-TZP surfaces and found higher wettability on modified surfaces compared to smooth, untreated specimens. The authors attributed this to the enhancement of surface energy caused by laser treatment [108]. Though still at an early stage of research, UV treatment of zirconia surfaces was reported to induce profound alterations in the physicochemical properties of the tested specimens. These changes were represented by a significant decrease in carbon content and conversion of the surface from hydrophobic to hydrophilic status. However, the exact mechanism behind such alterations and its impact on osteoconductivity are still to be investigated [109]. Coating the surface of Y-TZP implants with bioactive glass was also reported to accelerate bone healing and to improve the osseointegration process [110].

Numerous other *in vitro* studies investigated the influence of different surface modifications on osteoblast and fibroblast cell adhesion to zirconia implants, concluding that the surface roughness of zirconia improves initial bone healing and resistance to removal torque [111,112,113,114].

In concordance with the previous literature, a recent systematic review could not find a statistical difference in removal torque values and bone implant contact between zirconia and titanium implants in different animal studies [115].

## 5. Peri-Implant Soft Tissues around Zirconia and Titanium Implants

Recently, Mellinghoff [116] presented a review of literature on peri-implant soft tissue around zirconia implants, comparing the results with the experience from his own practice. The findings showed that zirconia implants and abutments provide a very good peri-implant soft tissue interface that achieves an irritation-free attachment. Various *in vivo* and *in vitro* investigations of soft tissue response around zirconia revealed comparable [117] or even better healing response, less inflammatory infiltrate and reduced plaque adhesion on zirconium oxide discs compared to conventionally pure titanium [118,119,120,121].

In a human *in vivo* study, Scarano *et al.* [119] quantified the percentage of surface coverage of titanium and zirconium oxide discs by bacteria and found a statistically significant difference between the two materials. The zirconium oxide surfaces showed a significant reduction in bacterial adhesion when compared to the titanium specimens. This could positively affect the health of peri-implant soft tissues as suggested by the authors. Another *in vivo* human study compared vascular endothelial growth factor (VEGF) and nitrous oxide synthase (NOS) expression, inflammatory infiltrate and microvessel density (MVD) in peri-implant soft tissue of titanium and zirconium healing caps. The results revealed higher values of VEGF, NOS, MVD and greater extension of inflammatory infiltrate with a subsequently higher rate of inflammation-associated processes in the titanium specimens compared to that of zirconium oxide specimens [120]. Moreover, an *in vivo* animal study analysed soft tissue responses to implant abutments made of titanium, ZrO_2_, Ti and Au-Pt alloy and found no difference in the soft tissue dimensions between Ti and ZrO_2_ abutments at two and five months of healing; however, a significant difference was found between the two materials and Au-Pt alloy [122]. In accordance, Kohal *et al.* [95] found no difference in soft tissue integration around rough titanium and zirconia implants in a monkey model.

In an *in vitro* and *in vivo* study, Rimondini *et al.* [118] compared oral bacterial colonization on the surfaces of disks fabricated from machined Grade 2 Ti and Y-TZP. Y-TZP was found to accumulate fewer bacteria than Ti and was suggested to be a promising material for abutment manufacturing. On the other hand, Lima *et al.* [123] and Al-Ahmad *et al.* [124] found that Ti and ZrO_2_ surfaces displayed similar biological properties in terms of protein adsorption, biofilm composition and bacterial adherence.

The attachment, growth behaviour and the genetic effect of human gingival fibroblasts (HGF) cultured on titanium and different zirconia surfaces (smooth and grooved) have also been investigated. HGFs showed comparable biological responses to both grooved zirconia ceramic and pure titanium surfaces [117].

## 6. Clinical Studies, Case Reports and Case Series on Zirconia Implants

A recent study analysed the survival and success of zirconia dental implants based on the available clinical data from case reports, prospective and retrospective clinical studies and randomized multicentre studies [125]. All of these studies, except one [126], reported the use of one-piece zirconia implants. The post-observation period ranged from one to five years with a reported survival rate of 74%–98% after 12–56 months and success rates between 79.6% and 91.6% after 6–12 months of prosthetic restoration. Nevertheless, the authors highlighted that there is a low-level of evidence to support the use of zirconia dental implants due to the limited period of observation and the number of participants in the included studies. Furthermore, they stressed the urgent need for well-conducted, long-term, randomized controlled trials to establish an evidence-based use of zirconia implants as an alternative to titanium implants [125].

A number of case reports demonstrated the use of one-piece zirconia implants with a roughened surface for the replacement of missing single, as well as multi-rooted teeth in either jaw with excellent aesthetic and functional outcomes after a follow-up period ranging from one to three years [127,128,129,130,131,132,133,134,135]. In the same context, the results of a prospective case series on the outcome of immediately provisionalised single-piece zirconia implants restoring single tooth gaps in the maxilla and mandible revealed comparable results to immediately restored titanium implants after 24 months of clinical function [136].

The success rate of one-piece zirconia implants with different surface treatments was evaluated in a number of prospective studies. The results revealed overall success rates of 92%–95% over follow-up periods ranging from 2.5 to five years, with excellent aesthetic and functional results. No mechanical complications were encountered, and it was suggested that zirconia implants might be a viable alternative to titanium implants for tooth replacement [137,138,139,140]. Similarly, a recent retrospective study revealed a three-year cumulative survival rate of 96.5% for endosseous zirconia implants in partially edentulous cases [141].

On the other hand, the findings of a cohort prospective study evaluating the clinical and radiographic outcomes of a one-piece zirconia oral implant for single tooth replacement could not confirm the previous results. The one-year follow-up showed a comparable survival rate for the immediately loaded ceramic and titanium implants. However, the increased radiographic bone loss of more than 2 mm around ceramic implants precluded its recommendation for clinical use [142]. In accordance were the results of a recent prospective case series that revealed increased radiographic bone loss (>2 mm) after one year around the one-piece zirconia implant system [143].

A multicentre randomized clinical trial, which evaluated immediate occlusal and non-occlusal loading of single zirconia implants, could not provide a conclusive answer. Alternatively, the conclusion of the study was that immediately loaded zirconia implants placed in post-extractive sites had higher failure rates than implants placed in healed sites [144].

Two recent prospective clinical studies reported the use of two-piece zirconia implants for the replacement of missing teeth in partially edentulous jaws. The results revealed possible clinical application of the two-piece system with emphasis on the need for more controlled and longer-term trials to confirm the presented data [145,146].

## 7. Discussion

This literature review attempted to explore the physical and mechanical properties of different dental implant materials and to correlate these properties with the designs and dimensions of the commercially available implants. Data, such as those previously discussed, enable the prediction of the long-term behaviour and the performance of any given dental implant and optimize the design of the next generation of dental implants.

Titanium is the most commonly used material for the fabrication of oral implants. This is supported by favourable mid- and long-term clinical outcomes [10,11,12]. The question of whether sensitivity and allergy to titanium implants is an issue of clinical relevance or not remains a topic to be investigated [23,25]. Titanium implants usually fail as a result of high cyclic loading resulting in peri-implant bone resorption; increased bending moments on implants and eventual metal fatigue and implant fracture [45,48,49,50]. With the introduction of improved strength Roxolid (Ti-Zr) narrow diameter implants, the array of dental implant indications is also increased. Implant therapy can be offered to patients with anatomically compromised situations, as well as to patients refusing extensive surgical procedures prior to implant rehabilitation [9,18]. However, possible mechanical problems with reduced diameter implants should not be disregarded, and sound clinical judgment is still mandatory before indicating these implants in posterior jaw regions [19].

The drive towards ceramic implants to satisfy the increasing aesthetic demands and metal-free request of some patients is fraught with compromise [147,148]. Zirconia remains a brittle ceramic material with a significant sensitivity to surface defects [78,79]. Therefore, strict quality control during the manufacturing process is a necessity to improve the clinical outcomes of zirconia implants [78]. All areas that can act as stress concentration sites should be avoided or minimized when designing zirconia implants. Sharp thread design, as well as internal line angles at the junction of threads with the implant body should be reduced. Thread depth should be also considered. Deep thread depths may interfere with bone clearance during the surgical implant placement, especially in the case of dense bone. This may generate unnecessary bending forces of the implant and eventually result in implant failure and fracture [78]. Thus, future studies should focus on evaluating optimal thread depth design for the next generation of zirconia implants. As occlusal load induced bending moments increase with bone loss, any rapid marginal bone loss should be investigated for possible mechanical complications, including fixture fracture [49,50]. The use of reduced diameter (3.25 mm) zirconia implants should be avoided [80]. Modifications of the surgical protocol may be necessary during the placement of zirconia implants in dense hard-type bone. Slight over-preparation of the osteotomy site is recommended to avoid the need for hand torqueing and, thus, the possible unfavourable bending forces on the implants [78]. With the absence of any studies correlating the LTD phenomena to the *in vivo* clinical performance of zirconia, this factor should not be disregarded [149]. From a histological and osseointegration perspective, zirconia can be recommended as a material for the fabrication of oral implants. All of the previously described animal studies revealed similar or even better bone growth onto zirconia as compared to titanium surfaces. Clinic-wise, there is no valid scientific data available in the literature to recommend the routine clinical use of zirconia implants; the quality of the clinical studies available in the literature is questionable. The majority of the studies are either case reports or case series with a limited number of participants and short-term follow-up periods.

Rapid prototyping or additive manufacturing is an emerging new technology, which should be explored for the possible fabrication of the next generation of both zirconia and titanium oral implants. Using rapid prototyping technology, a 3D custom-made layered structure can be constructed through computer-controlled extrusion of colloidal pastes, slurries or inks [150,151]. The benefit of this technique over the conventional milling method is that it is more economical with minimal material wastage. Any unused material can be reused for future processing. Multiple objects can be printed at one single time. In the case of zirconia, the material hardness is no longer an issue, as is the case with milling. Densely sintered zirconia is very hard, rendering the milling burs susceptible to wear and tool failure. Furthermore, fine details may be under-milled and the material is more prone to chipping, chattering and degradation of the overall mechanical properties of the material [150]. Briefly, rapid prototyping is a novel manufacturing technique that can allow the fabrication of custom-made implants that suit the specific anatomical situation of each individual patient in the material of choice and represents a field that should be explored.

## References

[1] Brånemark P.I., Hansson B.O., Adell R., Breine U., Lindström J., Hallén O., Ohman A. (1977). Osseointegrated implants in the treatment of the edentulous jaw. Experience from a 10-year period. Scand. J. Plast. Reconstr. Surg..

[2] Smith D.C. (1993). Dental implants: Materials and design considerations. Int. J. Prosthodont..

[3] Parr G.R., Gardner L.K., Toth R.W. (1985). Titanium: The mystery metal of implant dentistry. Dental materials aspect. J. Prosthet. Dent..

[4] Sykaras N., Lacopino A.M., Marker V.A., Triplett R.G., Woody R.D. (2000). Implant materials, designs, and surface topographies: Their effect on osseointegration. A literature review. Int. J. Oral Maxillofac. Implants.

[5] Williams D.E. (1981). Implants in dental and maxillofacial surgery. Biomaterials.

[6] Lemons J.E. (1990). Dental implants biomaterials. J. Am. Dent. Assoc..

[7] Craig R.G. (1993). Restorative Dental Materials.

[8] Sagomonyants K.B., Jarman-Smith M.L., Devine J.N., Aronow M.S., Gronowicz G.A. (2007). The *in vitro* response of human osteoblasts to polyetheretherketone (PEEK) substrates compared to commercially pure titanium. Biomaterials.

[9] Berner S., Dard M., Gottlow J., Molenberg A., Wieland M. (2009). Titanium-zirconium: A novel material for dental implants. Eur. Cells Mater..

[10] Adell R., Eriksson B., Lekholm U., Brånemark P.I., Jemt T. (1990). A long-term follow-up study of osseointegrated implants in the treatment of totally edentulous jaws. Int. J. Oral Maxillofac. Implants.

[11] Jemt T., Chai J., Harnett J., Heath M.R., Hutton J.E., Johns R.B., McKenna S., McNamara D.C., van Steenberghe D., Taylor R. (1996). A 5-year prospective multicenter follow-up report on overdentures supported by osseointegrated implants. Int. J. Oral Maxillofac. Implants.

[12] Niinomi M. (1998). Mechanical properties of biomedical titanium alloy. Mat. Sci. Eng. A.

[13] Wataha J.C. (1996). Materials for endosseous dental implants. J. Oral Rehabil..

[14] McCracken M. (1999). Dental implant materials: Commercially pure titanium and titanium alloys. J. Prosthodont..

[15] González J.E.G., Mirza-Rosca J.C. (1999). Study of the corrosion behavior of titanium and some of its alloys for biomedical and dental implant applications. J. Electroanal Chem..

[16] Bidez M.W., Misch C.F. (1992). Force transfer in implant dentistry: Basic concepts and principles. J. Oral Implantol..

[17] Odin G., Savoldelli C., Bouchard P.O., Tillier Y. (2010). Determination of Young’s modulus of mandibular bone using inverse analysis. Med. Eng. Phys..

[18] Grandin H.M., Berner S., Dart M. (2015). A review of titanium zirconium (TiZr) alloys for use in endosseous dental implants. Materials.

[19] Karl M., Krafft T., Kelly J.R. (2014). Fracture of a narrow-diameter Roxolid implant: Clinical and fractographic considerations. Int. J. Oral Maxillofac. Implants.

[20] Olmedo D.G., Paparella M.L., Brandizzi D., Cabrini R.L. (2010). Reactive lesions of peri-implant mucosa associated with titanium dental implants: A report of 2 cases. Int. J. Oral Maxillofac. Surg..

[21] Egusa H., Ko N., Shimazu T., Yatani H. (2008). Suspected association of an allergic reaction with titanium dental implants: A clinical report. J. Prosthet. Dent..

[22] Flatebø R.S., Johannessen A.C., Grønningsæter A.G., Bøe O.E., Gjerdet N.R., Grung B., Leknes K.N. (2006). Host response to titanium dental implant placement evaluated in a human oral model. J. Periodontol..

[23] Sicilia A., Cuesta S., Coma G., Arregui I., Guisasola C., Ruiz E., Maestro A. (2008). Titanium allergy in dental implant patients: A clinical study on 1500 consecutive patients. Clin. Oral Implants Res..

[24] Chaturvedi T.P. (2009). An overview of the corrosion aspect of dental implants (titanium and its alloys). Indian J. Dent. Res..

[25] Siddiqi A., Payne A.G.T., de Silva R.K., Duncan W.J. (2011). Titanium allergy: Could it affect dental implant integration?. Clin. Oral Implants Res..

[26] Javed F., Al-Hezaimi K., Almas K., Romanos G.E. (2013). Is titanium sensitivity associated with allergic reactions in patients with dental implants? A systematic review. Clin. Implant Dent. Relat. Res..

[27] Hallab N., Merritt K., Jacobs J.J. (2001). Metal sensitivity in patients with orthopaedic implants. J. Bone Joint Surg. Am..

[28] Frisken K.W., Dandie G.W., Lugowski S., Jordan G. (2002). A study of titanium release into body organs following the insertion of single threaded screw implants into the mandibles of sheep. Aust. Dent. J..

[29] Jacobs J.J., Skipor A.K., Black J., Urban R., Galante J.O. (1991). Release and excretion of metal in patients who have a total hip replacement component made of titanium- base alloy. J. Bone Joint Surg. Am..

[30] Witt J.D., Swann M. (1991). Metal wear and tissue response in failed titanium alloy total hip replacements. J. Bone Joint Surg. Br..

[31] Tamai K., Mitsumori M., Fujishiro S., Kokubo M., Ooya N., Nagata Y., Sasai K., Hiraoka M., Inamoto T. (2001). A case of allergic reaction to surgical metal clips inserted for postoperative boost irradiation in a patient undergoing breast conserving therapy. Breast Cancer.

[32] Thomas P., Bandl W.D., Maier S., Summer B., Przybilla B. (2006). Hypersensitivity to titanium osteosynthesis with impaired fracture healing, eczema, and T-cell hyper responsiveness *in vitro*: Case report and review of the literature. Contact Dermat..

[33] Haug R.H. (1996). Retention of asymptomatic bone plates used for orthognathic surgery and facial fractures. J. Oral Maxillofac. Surg..

[34] Schramm M., Pitto R.P., Willmann G., Zweymüller K. (2000). Clinical relevance of allergological tests in total hip joint replacement. Bioceramics in Hip Joint Replacement.

[35] Bass J.K., Fine H., Cisneros G.J. (1993). Nickel hypersensitivity in the orthodontic patient. Am. J. Orthod. Dentofac. Orthop..

[36] Mitchell D.L., Synnott S.A., van Dercreek J.A. (1990). Tissue reaction involving an intraoral skin graft and CP titanium abutments: A clinical report. Int. J. Oral Maxillofac. Implants.

[37] Du Preez L.A., Bütow K.W., Swart T.J. (2007). Implant failure due to titanium hypersensitivity/allergy?—Report of a case. SADJ.

[38] Müller K., Valentine-Thon E. (2006). Hypersensitivity to titanium: Clinical and laboratory evidence. Neuro. Endocrinol. Lett..

[39] Chaturvedi T.P. (2013). Allergy related to dental implant and its clinical significance. Clin. Cosmet. Investig. Dent..

[40] Balshi T.H. (1996). An analysis and management of fractured implants: A clinical report. Int. J. Oral Maxillofac. Implants.

[41] Tolman D.E., Laney W.R. (1992). Tissue integrated prosthesis complications. Int. J. Oral Maxillofac. Implants.

[42] Jemt T., Lekholm U. (1993). Oral implant treatment in posterior partially edentulous jaws: A 5-year follow-up report. Int. J. Oral Maxillofac. Implants.

[43] Zarb G., Schmitt A. (1990). The longitudinal clinical effectiveness of osseointegrated dental implants: The Toronto study. Part III: Problems and complications encountered. J. Prosthet. Dent..

[44] Piattelli A., Scarano A., Piattelli M., Vaia E., Matarasso S. (1998). Hollow implants retreived for fracture: A light and scanning electron microscope analysis of 4 cases. J. Periodontol..

[45] Piattelli A., Piattelli M., Scarano M., Montesani L. (1998). Light and scanning electron microscopic report of four fractured implants. Int. J. Oral Maxillofac. Implants.

[46] Eckert S.E., Meraw S.J., Cal E., Ow R.K. (2000). Analysis of incidence and associated factors with fractured implants: A retrospective study. Int. J. Oral Maxillofac. Implants.

[47] Green N.T., Machtei E.E., Horwitz J., Peled M. (2002). Fracture of dental implants: Literature review and report of a case. Implant Dent..

[48] Patterson E.A., Johns R.B. (1992). Theoretical analysis of the fatigue life of fixture screws in oseeointegrated dental implants. Int. J. Oral Maxillofac. Implants.

[49] Morgan M.J., James D.F., Pilliar R.M. (1993). Fractures of fixture component of an oseeointegrated implant. Int. J. Oral Maxillofac. Implants.

[50] Virdee P., Bishop K. (2007). A review of the aetiology and management of fractured dental implants and a case report. Br. Dent. J..

[51] Yokoyama K., Ichikawa T., Murakami H., Miyamoto Y., Asaoka K. (2002). Fracture mechanisms of retrieved titanium screw thread in dental implant. Biomaterials.

[52] Lacefield W.R. (1998). Current status of ceramic coatings for dental implants. Implant Dent..

[53] De Groot K., Wolke J.C.G., Jansen J.A. (1994). State of the art: Hydroxylapatite coatings for dental implants. J. Oral Implant..

[54] Morris H.F., Ochi S., Spray J.R., Olson J.W. (2000). Periodontal-type measurements associated with hydroxyapatite-coated and non-HA-coated implants: Uncovering to 36 months. Ann. Periodontol..

[55] Barrere F., van der Valk C.M., Meijer G., Dalmeijer R.A., de Groot K., Layrolle P. (2003). Osteointegration of biomimetic apatite coating applied onto dense and porous metal implants in femurs of goats. J. Biomed. Mater. Res..

[56] Le Guéhennec L., Soueidan A., Layrolle P., Amouriq Y. (2007). Surface treatments of titanium dental implants for rapid osseointegration. J. Dent. Mater..

[57] Wheeler S. (1996). Eight-year clinical retrospective study of titanium plasma-sprayed and hydroxyapatite-coated cylinder implants. Int. J. Oral Maxillofac. Implants.

[58] Chang Y.-L., Lew D., Park J.B., Keller J.C. (1999). Biomechanical and morphometric analysis of hydroxyapatite-coated implants with varying crystallinity. J. Oral Maxillofac. Surg..

[59] Tinsley D., Watson C., Russell J. (2001). A comparison of hydroxyapatite coated implant retained fixed and removable mandibular prostheses over 4 to 6 years. Clin. Oral Implants Res..

[60] Kohal R.J., Att W., Bächle M., Butz F. (2008). Ceramic abutments and ceramic oral implants. An update. Periodontol. 2000.

[61] Black J., Hastings G. (1998). Oxide bioceramics: Inert ceramic materials in medicine and dentistry. Handbook of Biomaterial Properties.

[62] Denry I., Kelly J.R. (2008). State of the art of zirconia for dental applications. Dent. Mater..

[63] Piconi C., Maccauro G. (1999). Zirconia as a ceramic biomaterial. Biomaterials.

[64] Kohal R.J., Klaus G., Strub J.R. (2006). Zirconia-implant supported all-ceramic crowns withstand long-term load: A pilot investigation. Clin. Oral Implants Res..

[65] Kohal R.J., Wolkewitz M., Tsakona A. (2011). The effects of cyclic loading and preparation on the fracture strength of zirconium-dioxide implants: An *in vitro* investigation. Clin. Oral Implants Res..

[66] Silva N., Coelho P.G., Fernandes C., Navarro J.M., Dias R.A., Thompson V.P. (2009). Reliability of one-piece ceramic implant. Biomed. Mater. Res. B App. Biomater..

[67] Kohal R.J., Finke H.C., Klaus G. (2009). Stability of prototype two piece zirconia and titanium implants after artificial aging: An *in vitro* pilot study. Clin. Implant Dent. Relat. Res..

[68] Garvie R., Hannink R., Pascoe R. (1975). Ceramic steel?. Nature.

[69] Chevalier J., Loh J., Gremillard L., Meille S., Adolfson E. (2011). Low-temperature degradation in zirconia with a porous surface. Acta Biomater..

[70] Lawson S. (1995). Environmental degradation of zirconia ceramics. J. Eur. Ceram. Soc..

[71] Chevalier J. (2006). What future for zirconia as a biomaterial?. Biomaterials.

[72] Watanabe M., Iio S., Fukuura I., Claussen N., Ruhle M., Heuer A.H. (1984). Ageing behaviour of Y-TZP. Science and Technology of Zirconia II, Advances in Ceramics.

[73] Att W., Grigoriadou M., Strub J.R. (2007). ZrO_2_ three-unit fixed partial dentures: Comparison of failure load before and after exposure to a mastication simulator. J. Oral Rehabil..

[74] Alghazzawi T.F., Lemons J., Liu P.R., Essig M.E., Bartolucci A.A., Janowski G.M. (2012). Influence of low temperature environmental exposure on the mechanical properties and structural stability of dental zirconia. J. Prosthodont..

[75] Samodurova A., Andraž K., Swain M.V., Tomaž K. (2014). The combined effect of alumina and silica co-doping on the ageing resistance of 3Y-TZP bioceramics. Acta Biomater..

[76] Piconi C., Burger W., Richter H.G., Cittadini A., Maccauro G., Covacci V., Bruzzese N., Ricci G.A., Marmo E. (1998). Y-TZP for artificial joint replacements. Biomaterials.

[77] Lee S.K., Tandon R., Ready M.J., Lawn B.R. (2000). Scratch damage on zirconia ceramics. J. Am. Cream. Soc..

[78] Osman R.B., Ma S., Duncan W., de Silva R.K., Siddiqi A., Swain M.V. (2013). Fractured Zirconia implants and related implant designs: Scanning electron microscopy analysis. Clin. Oral Implants Res..

[79] Zhang Y., Sailer I., Lawn B.R. (2013). Fatigue of dental ceramics. J. Dent..

[80] Gahlert M., Burtscher D., Grunert I., Kniha H., Steinhauser E. (2012). Failure analysis of fractured dental zirconia implants. Clin. Oral Implants Res..

[81] Sanon C., Chevalier J., Douillard T., Cattani-Lorente M., Scherrer S.S., Gremillard L. (2015). A new testing protocol for zirconia dental implants. Dent. Mater..

[82] Ruiz L., Readey M.J. (1996). Effect of heat-treatment on grain size, phase assemblage, and mechanical properties of 3 mol % Y-TZP. J. Am. Ceram. Soc..

[83] Heuer A.H., Claussen N., Kriven W.M., Ruhle M. (1982). Stability of tetragonal ZrO_2_ particles in ceramic matrices. J. Am. Ceram. Soc..

[84] Cottom B.A., Mayo M.J. (1996). Fracture toughness of nanocrystalline ZrO_2_–3 mol % Y_2_O_3_ determined by Vickers indentation. Scr. Mater..

[85] Filser F., Kocher P., Gauckler L.J. (2003). Net-shaping of ceramic components by direct ceramic machining. Assembly Autom..

[86] Ross I.M., Rainforth W.M., McComb D.W., Scott A.J., Brydson R. (2001). The role of trace additions of alumina to yttria-tetragonal zirconia polycrystal (Y-TZP). Scr. Mater..

[87] Li L.F., Watanabe R. (1997). Influence of a small amount of Al_2_O_3_ addition on the transformation of Y_2_O_3_-partially stabilized ZrO_2_ during annealing. J. Mater. Sci..

[88] Tsubakino H., Nozato R., Hamamoto M. (1991). Effect of alumina addition on the tetragonal-to-monoclinic phase transformation in zirconia–3 mol % yttria. J. Am. Ceram. Soc..

[89] Guazzato M., Albakry M., Quach L., Swain M.V. (2004). Influence of grinding, sandblasting, polishing and heat treatment on the flexural strength of a glass-infiltrated alumina-reinforced dental ceramic. Biomaterials.

[90] Guazzato M., Albakry M., Swain M.V., Ringer S.P. (2003). Microstructure of alumina-and alumina/zirconia-glass infiltrated dental ceramics. Bioceramics.

[91] Guazzato M., Albakry M., Ringer S.P., Swain M.V. (2004). Strength, fracture toughness and microstructure of a selection of all-ceramic materials. Part II. Zirconia-based dental ceramics. Dent. Mater..

[92] Pabst W., Havrda E., Gregorová E., Krčmová B. (2000). Alumina toughened zirconia made by room temperature extrusion of ceramic pastes. J. Am. Ceram. Soc..

[93] Akagawa Y., Ichikawa Y., Nikai H., Tsuru H. (1993). Interface histology of unloaded and early loaded partially stabilized zirconia endosseous implant in initial bone healing. J. Prosthet. Dent..

[94] Akagawa Y., Hosok R., Sato Y., Kamayama K. (1998). Comparison between freestanding and tooth-connected partially stabilized zirconia implants after two years function in monkeys: A clinical and histologic study. J. Prosthet. Dent..

[95] Kohal R.J., Weng D., Bӓchle M., Strub J.R. (2004). Loaded custom-made zirconia and titanium implants show similar osseointegration: An animal experiment. J. Periodontol..

[96] Dubruille J.H., Viguier E., Le Naour G., Dubruille M.T., Auriol M., Le Charpentier Y. (1999). Evaluation of combinations of titanium, zirconia, and alumina implants with 2 bone fillers in the dog. Int. J. Oral Maxillofac. Implants.

[97] Scarano A., Di Carlo F., Quaranta M., Piattelli A. (2003). Bone response to zirconia ceramic implants: An experimental study in rabbits. J. Oral Implantol..

[98] Depprich R., Zipprich H., Ommerborn M., Mahn E., Lammers L., Handschel J., Naujoks C., Wiesmann H.P., Kübler N.R., Meyer U. (2008). Osseointegration of zirconia implants: An SEM observation of the bone-implant interface. Head Face Med..

[99] Hoffmann O., Angelov N., Gallez F., Jung R.E., Weber F.E. (2008). The zirconia implant-bone interface: A preliminary histologic evaluation in rabbits. Int. J. Oral Maxillofac. Implants.

[100] Gahlert M., Roehling S., Sprecher C.M., Kniha H., Milz S., Bormann K. (2012). *In vivo* performance of zirconia and titanium implants: A histomorphometric study in mini pig maxillae. Clin. Oral Implants Res..

[101] Schultze-Mosgau S., Schliephake H., Radespiel-Troger M., Neukam F.W. (2000). Osseointegration of endodontic endosseous cones: Zirconium oxide vs titanium. Oral Surg. Oral Med. Oral Pathol. Oral Radiol. Endod..

[102] Sennerby L., Dasmah A., Larsson B., Iverhed M. (2005). Bone tissue response to surface modified zirconia implants: A histomorphometric and removal torque study in the rabbit. Clin. Implant Dent. Relat. Res..

[103] Rocchietta I., Fontana F., Addis A., Schupbach P., Simon M. (2009). Surface-modified zirconia implants: Tissue response in rabbits. Clin. Oral Implants Res..

[104] Gahlert M., Gudehus T., Eichhorn S., Steinhauser E., Kniha H., Erhardt W. (2007). Biomechanical and histomorphometric comparison between zirconia implants with varying surface textures and a titanium implant in the maxilla of miniature pigs. Clin. Oral Implants Res..

[105] Schliephake H., Hefti T., Schlottig F., Gédet P., Staedt H. (2010). Mechanical anchorage and peri-implant bone formation of surface-modified zirconia in minipigs. J. Clin. Periodontol..

[106] Bächle M., Butz F., Hübner U., Bakalinis E., Kohal R.J. (2007). Behavior of CAL72 osteoblast-like cells cultured on zirconia ceramics with different surface topographies. Clin. Oral Implants Res..

[107] Hempel U., Hefti T., Kalbacova M., Wolf-Brandstetter C., Dieter P., Schlottig F. (2010). Response of osteoblast-like SAOS-2 cells to zirconia ceramics with different surface topographies. Clin. Oral Implants Res..

[108] Hao L., Lawrence J., Chian K.S. (2005). Osteoblast cell adhesion on a laser modified zirconia based bioceramic. J. Mater. Sci. Mater. Med..

[109] Tuna T., Wein M., Swain M., Fischer J., Att W. (2015). Influence of ultraviolet photofunctionalization on the surface characteristics of zirconia-based dental implant materials. Dent. Mater..

[110] Aldini N.N., Fini M., Giavaresi G., Martini L., Dubini B., Ponzi-Bossi M.G., Rustichelli F., Krajewski A., Ravaglioli A., Mazzocchi M. (2004). Osteointegration of bioactive glass coated and uncoated zirconia in osteopenic bone: An *in vivo* experimental study. J. Biomed. Mater. Res..

[111] Ferguson S.J., Langhoff J.D., Voelter K., von Rechenberg B., Scharnweber D., Bierbaum S., Schnabelrauch M., Kautz A.R., Frauchiger V.M., Mueller T.L. (2008). Biomechanical comparison of different surface modifications for dental implants. Int. J. Oral Maxillofac. Implants.

[112] Payer M., Lorenzoni M., Jakse N., Kirmeier R., Dohr G., Stopper M., Pert C. (2010). Cell growth on different zirconia and titanium surface textures: A morphologic *in vitro* study. Z. Zahnӓrztl. Implants.

[113] Zinelis S., Thomas A., Syres K., Silikas N., Eliades G. (2010). Surface characterization of zirconia dental implants. Dent. Mater..

[114] Park Y.S., Chung S.H., Shon W.J. (2013). Peri-implant bone formation and surface characteristics of rough surface zirconia implants manufactured by powder injection molding technique in rabbit tibiae. Clin. Oral Implants Res..

[115] Manzano G., Herrero L.R., Montero J. (2014). Comparison of clinical performance of zirconia implants and titanium implants in animal models: A systematic review. Int. J. Oral Maxillofac. Implants.

[116] Mellinghoff J. (2010). Quality of the peri-implant soft tissue attachment of zirconia implants-abutments. Z. Zahnärztl. Implants.

[117] Pae A., Lee H., Kim H.S., Kwon Y.D., Woo Y.H. (2009). Attachment and growth behavior of human gingival fibroblasts on titanium and zirconia ceramic surfaces. Biomed. Mater..

[118] Rimondini L., Cerroni L., Carrassi A., Torricelli P. (2002). Bacterial colonization of zirconia ceramic surfaces: An *in vitro* and *in vivo* Study. Int. J. Oral Maxillofac. Implants.

[119] Scarano A., Piattelli M., Caputi S., Favero G.A., Piattelli A. (2004). Bacterial adhesion on commercially pure titanium and zirconium oxide discs: An *in vivo* human study. J. Periodontol..

[120] Degidi M., Artese L., Scarano A., Perrotti V., Gehrke P., Piattelli A. (2006). Inflammatory infiltrate, microvessel density, nitric oxide synthase expression, vascular endothelial growth factor expression, and proliferative activity in peri-implant soft tissues around titanium and zirconium oxide healing caps. J. Periodontol..

[121] Größner-Schreiber B., Herzog M., Hedderich J., Dück A., Hannig M., Griepentrog M. (2006). Focal adhesion contact formation by fibroblasts cultured on surface-modified dental implants: An *in vitro* study. Clin. Oral Implants Res..

[122] Welander M., Abrahamsson I., Berglundh T. (2008). The mucosal barrier at implant abutments of different materials. Clin. Oral Implants Res..

[123] Lima E.M.C.X., Koo H., Vacca-Smith A.M., Rosalen P.L., Del Bel Cury A.A. (2008). Adsorption of salivary and serum proteins, and bacterial adherence on titanium and zirconia ceramic surfaces. Clin. Oral Implants Res..

[124] Al-Ahmad A., Wiedmann-Al-Ahmad M., Faust J., Bӓchle M., Follo M., Wolkewitz M., Hannig C., Hellwig E., Carvalho C., Kohal R. (2010). Biofilm formation and composition on different implant materials *in vivo*. J. Biomed. Mater. Res. B Appl. Biomater..

[125] Depprich R., Naujoks C., Ommerborn M., Schwarz F., Kübler N.R., Handschel J. (2014). Current findings regarding zirconia implants. Clin. Implant Dent. Relat. Res..

[126] Nevins M., Camelo M., Nevins M.L., Schupbach P., Kim D.M. (2011). Pilot clinical and histologic evaluations of a two-piece zirconia implant. Int. J. Periodontics Restor..

[127] Kohal R.J., Klaus G. (2004). A zirconia implant-crown system: A case report. Int. J. Periodontics Restor..

[128] Oliva J., Oliva X., Oliva J.D. (2008). Ovoid zirconia implants: Anatomic design for premolar replacement. Int. J. Periodontics Restor..

[129] Oliva J., Oliva X., Oliva J.D. (2008). Zirconia implants and all ceramic restorations for esthetic replacement of the maxillary central incisors. Eur. J. Esthet. Dent..

[130] Oliva X., Oliva J., Oliva J.D. (2008). Replacement of congenitally missing maxillary permanent canine with a zirconium oxide dental implant and crown. A case report from an ongoing clinical study. Oral Surg..

[131] Pirker W., Kocher A. (2008). Immediate, non-submerged, root-analogue zirconia implant in single tooth replacement. Int. J. Oral Maxillofac. Surg..

[132] Pirker W., Kocher A. (2011). Root analog zirconia implants: True anatomical design for molar replacement-A case report. Int. J. Periodontics Restor..

[133] Aydin C., Yilmaz H., Ata S.O. (2010). Single-tooth zirconia implant located in anterior maxilla. N. Y. State Dent. J..

[134] Pirker W., Wiedemann D., Lidauer A., Kocher A. (2011). Immediate, single stage, truly anatomic zirconia implant in lower molar replacement: A case report with 2.5 years follow-up. Int. J. Oral Maxillofac. Surg..

[135] Borgonovo A.E., Corrocher G., Dolci M., Censi R., Vavassori V., Maiorana C. (2013). Clinical evaluation of zirconium dental implants placed in esthetic areas: A case series study. Eur. J. Esthet. Dent..

[136] Payer M., Arnetzl V., Kirmeier R., Koller M., Arnetzl G., Jakse N. (2013). Immediate provisional restoration of single-piece zirconia implants: A prospective case series—Results after 24 months of clinical function. Clin. Oral Implants Res..

[137] Pirker W., Kocher A. (2009). Immediate, non-submerged, root-analogue zirconia implants placed into single-rooted extraction sockets: 2-year follow-up of a clinical study. Int. J. Oral Maxillofac. Surg..

[138] Oliva J., Oliva X., Oliva J. (2010). Five year success rate of 831 consecutively placed zirconia dental implants in humans: A comparison of three different rough surfaces. Int. J. Oral Maxillofac. Implants.

[139] Borgonovo A.E., Fabbri A., Vavassori V., Censi R., Maiorana C. (2012). Multiple teeth replacement with endosseous one-piece yttrium-stabilized zirconia dental implants. Med. Oral Patol. Oral Cir. Bucal.

[140] Borgonovo A.E., Censi R., Vavassori V., Dolci M., Josè Luis C.-G., Ruiz R.A.D., Maiorana C. (2013). Evaluation of the success criteria for zirconia dental implants: A four-year clinical and radiological study. Int. J. Dent..

[141] Brüll F., van Winkelhoff A., Cune M.S. (2014). Zirconia dental implants: A clinical, radiographic, and microbiologic evaluation up to 3 years. Int. J. Oral Maxillofac. Implants.

[142] Kohal R.J., Knauf M., Larsson B., Sahlin H., Butz F. (2012). One-piece zirconia oral implants: One-year results from a prospective cohort study. 1. Single tooth replacement. J. Clin. Periodontol..

[143] Kohal R.-J., Patzelt S.B.M., Butz F., Sahlin H. (2013). One-piece zirconia oral implants: One-year results from a prospective case series. 2. Three-unit fixed dental prosthesis (FDP) reconstruction. J. Clin. Periodontol..

[144] Cannizzaro G., Torchio C., Felice P., Leone M., Esposito M. (2010). Immediate occlusal *versus* non-occlusal loading of *single* zirconia implants. A multicentre pragmatic randomized clinical trial. Eur. J. Oral Implantol..

[145] Cionca N., Müller N., Mombelli A. (2014). Two-piece-zirconia implants supporting all-ceramic crowns. A prospective clinical study. Clin. Oral Implants Res..

[146] Payer M., Heschl A., Koller M., Arnetzl G., Lorenzoni M., Jakse N. (2014). All-ceramic restoration of zirconia two-piece implants—A randomized controlled clinical trial. Clin. Oral Implants Res..

[147] Osman R.B., Morgaine K.C., Duncan W., Swain M.V., Ma S. (2014). Patients’ perspectives on zirconia and titanium implants with a novel distribution supporting maxillary and mandibular overdentures: A qualitative study. Clin. Oral Implants Res..

[148] Osman R.B., Swain M.V., Atieh M., Ma S., Duncan W. (2014). Ceramic implants (Y-TZP): Are they a viable alternative to titanium implants for the support of overdentures?A randomized clinical trial. Clin. Oral Implants Res..

[149] Silva R.F.A., Sailer I., Zhang Y., Coelho P.G., Guess P.C., Zembic A., Kohal R.J. (2010). Performance of zirconia for dental healthcare. Materials.

[150] Abduo J., Lyons K., Bennamoun M. (2014). Trends in computer-aided manufacturing in prosthodontics: A review of the available streams. Int. J. Dent..

[151] Wätjen A.M., Gingter P., Kramer M., Telle R. (2014). Novel prospects and possibilities in additive manufacturing of ceramics by means of direct inkjet printing. Adv. Mech. Eng..

